# Supplementary material for: Self-sampling monkeypox virus testing in high-risk populations, asymptomatic or with unrecognized Mpox, in Spain
Source: Nat Commun. 2023 Oct 2;14:5998. doi: 10.1038/s41467-023-40490-9 (PMC10545734; doi:10.1038/s41467-023-40490-9)
Supplement: Supplementary file 1 — Supplementary Information [file 41467_2023_40490_MOESM1_ESM.pdf]

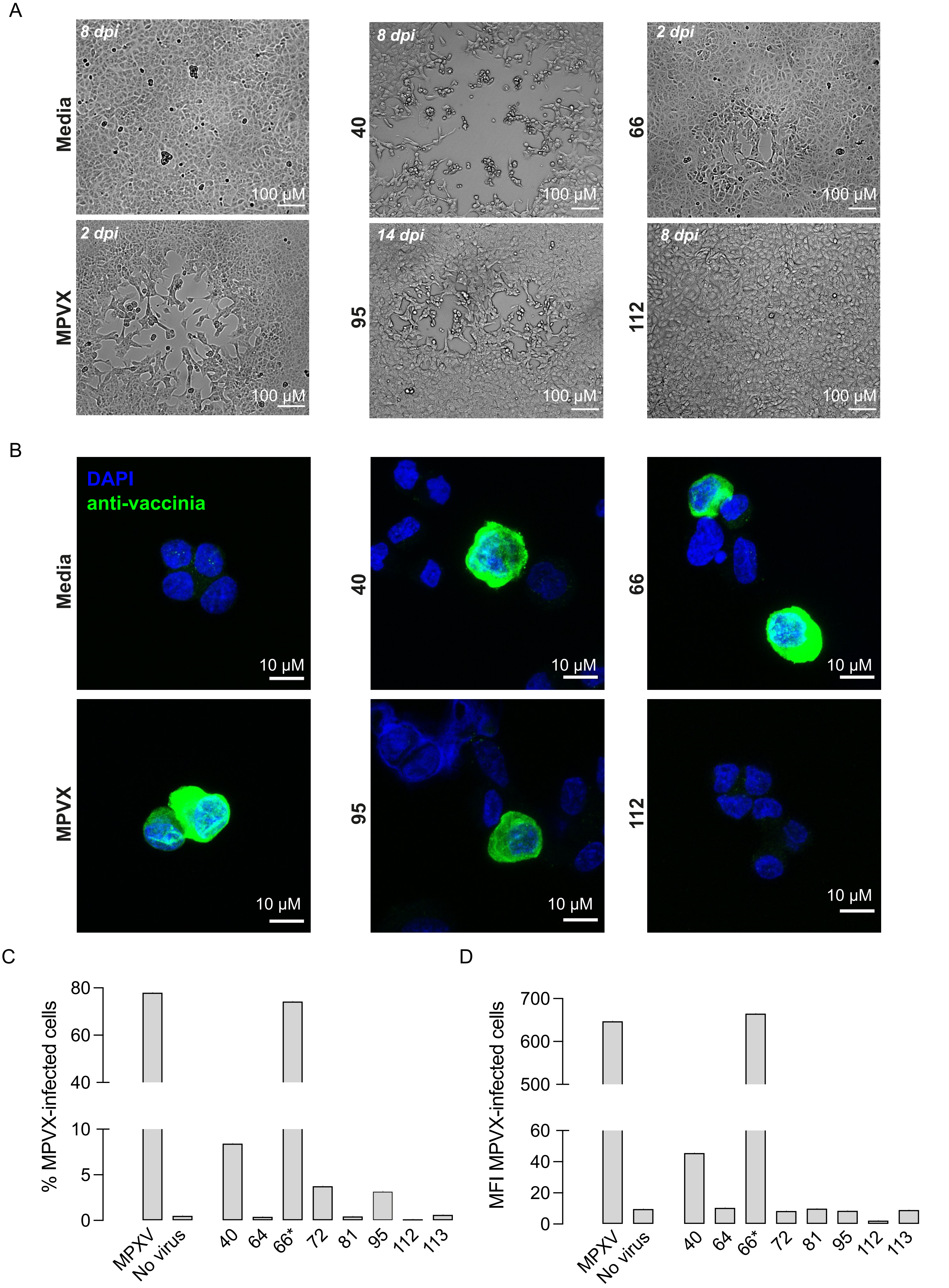

Supplementary Figure 1

Supplementary Figure 1: Representative results from samples assayed to isolate infectious MPVX. A. Optical microscopy images of Vero E6 cultures inoculated with swab samples. Images were taken at the day post infection (dpi) indicated in the top left part of the images. Scale bars correspond to 100 µm. Viral isolation was done only once due to sample availability B. Confocal microscopy images of cells where swab samples were grown after intracellular staining with the anti-vaccinia pAb revealed with an Alexa 488 secondary antibody from the cultures. DAPI staining is shown in blue (nuclei) and α-Vaccinia is shown in green. Scale bars correspond to 20 µm. Viral isolation was done only once due to sample availability C. Percentage of positive cells detected by FACS after intracellular staining as described in B. Sample with asterisk denotes that it had to be sub-cultured again as primary culture exceeded 95 % of CPE which precluded proper FACS analysis. D. Mean fluorescence intensity of positive cells detected by FACS after intracellular staining as described in B. Sample with asterisk had to be sub-cultured. Source data of C and D are provided as a Source Data file.

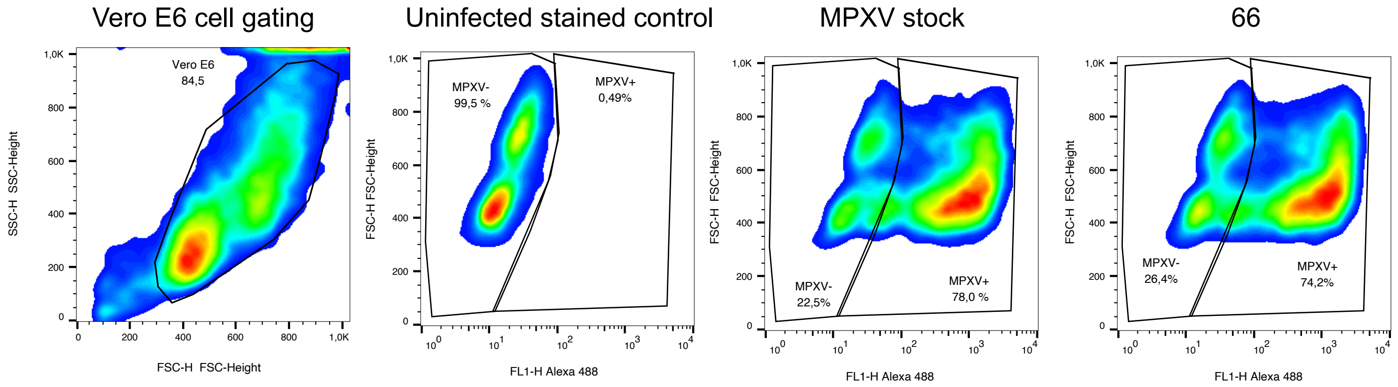

Supplemental Figure 2

Supplementary Figure 2: Gating strategy of Vero E6 cells FACS analysis shown in Supplemental Figures 1 C-D. Panels show gating of living cells, non-infected cells stained and used to set up the boundaries of positivity, a positive control infected with an isolated viral stock, and a positive isolated sample (66).

STOP Mpox

ID number:____________________(*Here you have to put one of the labels on the envelope).*
Date of questionnaire:_____/_____/__________(*DD / MM / YYYY)*

A. You are:

- ☐ cis-male *(my assigned gender and assigned sex match)*
- ☐ Trans woman
- ☐ Non-Binary Person
- ☐ Other, specify:______________________________________

B. You define yourself as...

- ☐ Gay
- ☐ Straight
- ☐ Bisexual
- ☐ Another sexual option, specify:____________________________

C. What is your date of birth? ____/_____/__________
DD /MM/ YYYYY

(This study is aimed exclusively at GBHSH and trans women 18 years of age or older).

D. Where were you born?

- ☐ Spain
- ☐ Other, specify:____

➔ **Year of arrival in** Spain: _______________________

PART A. SOCIODEMOGRAPHIC DATA

1. **What is the highest level of education you have completed?**
   - *a.* I have not completed any level of education *(pass question 2).*
   - *b.* Primary education (*skip question 2)*
   - *c.* Secondary education *(skip question 2)*
   - *d.* Intermediate or higher vocational training *(pass question 2)*
   - *e.* Bachelor's degree, bachelor's degree or diploma *(pass question 2)*
   - *f.* Postgraduate studies (master's, doctorate, etc.) *(skip question 2)*
   - *g.* DK/NA (*skip question 2)*

2. **On average, what is your monthly income?**
   - *a.* I currently do not receive any income *(skip question 3).*
   - *b.* Less than 500 euros *(pass question 3)*
   - *c.* 500 - 749 euros *(pass question 3)*
   - *d.* 750 - 999 euros *(pass question 3)*
   - *e.* 1000 1249 euros *(pass question 3)*
   - *f.* 1250 1499 euros *(skip question 3)*
   - *g.* 1500 - 1749 euros *(pass question 3)*
   - *h.* 1750 -1999 euros *(pass question 3)*
   - *i.* 2000 - 4000 euros *(pass question 3)*

j. More than 4,000 euros *(pass question 3)*
k. I prefer not to say *(pass question 3).*
l. DK/NA *(skip question 3)*

3. How many inhabitants does the city or town where you live have?

a. More than one million inhabitants *(pass question 4)*
b. Between 500,000-999,999 inhabitants *(pass question 4)*
c. Between 100,000-499,999 inhabitants *(pass question 4)*
d. Between 49,000 -99,999 inhabitants *(skip question 4)*
e. Between 10,000 and 49,000 inhabitants *(skip question 4)*
f. Less than 10,000 inhabitants *(pass question 4)*
g. DK/NA. (*skip question 4)*

4. Number of people living with you (in your household, including yourself):
______________*(skip question 5)*

5.* Who do you live with (*you can check more than one answer)?

- ☐ Alone *(pass question 6)*
- ☐ Couple/s *(skip question 6)*
- ☐ Child(ren) *(skip question 6)*
- ☐ Parents and sibling or only with parents (one or both) *(skip question 6)*
- ☐ Other relatives (uncle/uncles, cousins) *(skip question 6)*
- ☐ Friend(s) or roommate(s) *(skip question 6)*
- ☐ I live in a student residence *(pass question 6).*
- ☐ I live on the street (in an ATM, park, car) (*skip question 6).*
- ☐ I live in a shelter or foster home *(pass question 6).*
- ☐ Other (specify):____________________*(skip question 6)*
- ☐ DK/NA *(skip question 6)*

PART B. POSSIBLE EXPOSURES

6. Have you had contact with domestic animals at your usual residence or place of work (exotic animals are excluded)?

a. Yes *(skip question 7)*
b. No *(skip question 7)*
c. DK/NA *(skip question 7)*

7. Have you had contact with exotic animals in your usual residence or place of work?

By exotic animals we refer to species found outside their original or native habitat.

a. Yes *(skip question 8)*
b. No *(skip question 8)*
c. DK/NA *(skip question 8)*

8. **Have you traveled in the last 30 days?**
   a. Yes *(skip question 9)*
   b. No *(skip question 10)*
   c. DK/NA *(skip question 10)*
9. **Place(s) and period(s) of travel:**
   - Country:___________*(skip question 10)*
   - Municipality:_____________*(skip question 10)*
   - Round trip date:__________________*(pass question 10)*
10. **Have you had a risk exposure to Mpox during your working hours (needle stick, laboratory work, contact with potentially contaminated material, healthcare professional without PPE) in the last 30 days?**
   a. Yes *(skip question 11)*
   b. No *(skip question 11)*
   c. DK/NA *(skip question 11)*
11. **Have you had contact with a case of Mpox in the last 30 days (it excludes occupational exposures)?**
   a. Yes *(skip question 12)*
   b. No *(skip question 13)*
   c. DK/NA *(skip question 13)*
12. **What type of contact did you have with that person(s) (*Check all that you consider):***
   - ☐ Person I care for *(skip question 13)*
   - ☐ Sexual contact (touching, masturbation, oral sex, vaginal or anal penetration with or without ejaculation) (*skip question 13).*
   - ☐ Shared food, utensils, or dishes *(skip question 13).*
   - ☐ Shared clothing *(skip question 13)*
   - ☐ Towels or bedding shared at home or elsewhere *(skip question 13)*
   - ☐ We have gone on a trip together *(pass question 13)*
   - ☐ Shared bathrooms (sinks, showers) either in the home or elsewhere *(skip question 13).*
   - ☐ Physical contact (face to face, kissing, shaking hands, hugging...) *(skip question 13).*
   - ☐ Other (describe):_______________________*(skip question 13)*
   - ☐ DK/NA *(skip question 13)*

PART C. SEXUAL PRACTICES

13. **In the last 30 days, have you had sex?**
   By sex we mean any type of sexual contact: touching, masturbation, oral sex, vaginal or anal penetration with or without ejaculation.

   a. Yes *(skip question 14)*
   b. No *(skip question 21)*
   c. DK/NA *(skip question 21)*

14. **If you have had sex in the last 30 days, with how many people of each gender and/or sex?**
    a. Cis women. Number:_____ *(pass question 15)*
    b. Cis men. Number:________ *(pass question 15)*
    c. Trans women. Number:_____ *(skip question 15)*
    d. Trans men. Number:________ *(skip question 15)*
    e. Non-binary people. Number:______ *(pass question 15)*
    f. Unknown. Number:_______ *(pass question 15)*
    g. DK/NA *(skip question 15)*
15. **With how many men or trans women have you had receptive ("passive") ANAL intercourse in the last 30 days?**
    a. I have not played a receptive ("passive") person in the last 30 days *(skip question 16).*
    b. With 1 *(pass question 16)*
    c. 2-4 *(skip question 16)*
    d. 5-9 *(skip question 16)*
    e. 10-20 *(pass question 16)*
    f. More than 20 pairs *(pass question 16)*
    g. DK/NA *(skip question 16)*
16. **With how many men or trans women have you had insertive ("active") ANAL intercourse in the last 30 days?**
    a. I have not been an insertive ("active") in the last 30 days *(skip question 17).*
    b. With 1 *(skip question 17)*
    c. 2-4 *(skip question 17)*
    d. 5-9 *(skip question 17)*
    e. 10-20 *(pass question 17)*
    f. More than 20 pairs *(pass question 17)*
    g. DK/NA *(skip question 17)*

17. **Have you engaged in or have you ever engaged in the following sexual practices (Check all that apply):**
(skip question 18)

	YES	NO	DK/NA
Mutual masturbation	□	□	□
You have performed oral sex (sucking cock or pussy).	□	□	□
Have you ever had oral sex (sucking cock or pussy)?	□	□	□
You have made a "black kiss" (eat ass).	□	□	□
You have been "black kissed" (eat ass)	□	□	□
Vaginal penetration (insertive)	□	□	□
Vaginal penetration (receptive)	□	□	□
Double penetration in vagina and anus (sandwich)	□	□	□
Double or triple anal penetration	□	□	□
Fist/fisting/fist fucking by anus and/or vagina	□	□	□
'Golden rain' (pissing on other(s), drinking pee).	□	□	□
Scat (e.g., play with/eat feces)	□	□	□
Using and/or sharing sex toys (e.g., dildos, Chinese or Thai balls...).	□	□	□
Threesome	□	□	□
Group sex, gangbang	□	□	□
Another__________	□	□	□

18. **In the last 30 days, with your sexual contacts, how often did you use condoms when you had penetrative sex?**
    a. Never *(skip question 19)*
    b. Less than half *(pass question 19)*
    c. About half *(pass question 19)*
    d. More than half *(pass question 19)*
    e. Always *(skip question 19)*
    f. Not applicable because I have not had penetrative sex *(skip question 19).*
    g. DK/NA *(skip question 19)*
19. **Did you apply any products (e.g., massage oils, lubes, creams, sprays, gels, enemas) to each other's skin or to yourself during or after sexual intercourse in the last 30 days?**
    a. Yes, specify:________________*(skip question 20)*
    b. No *(skip question 20)*
    c. DK/NA *(skip question 20)*

20. Where did you meet your partners with whom you had sex in the last 30 days?
(Check all that you consider)

- ☐ Organized groupings (LGTBI+ organizations, sports club, choir, professional network meetings, etc.) *(skip question 21)*
- ☐ Cafes or bars *(skip question 21)*
- ☐ Discotheques *(skip question 21)*
- ☐ Darkroom in bars or nightclubs *(skip question 21)*
- ☐ Sex Clubs *(skip question 21)*
- ☐ Saunas *(skip question 21)*
- ☐ Sex-shops *(skip question 21)*
- ☐ Gym *(skip question 21)*
- ☐ Chill out/Chemsex session/sex with chems in private homes or hotels *(skip question 21)*
- ☐ Cruising areas (park, forest, public toilets) (*skip question 21)*
- ☐ Gay dating websites or apps (e.g. Grindr, Scruff,...)*(skip question 21).*
- ☐ General websites or apps (e.g. Tinder) *(skip question 21).*
- ☐ Social networking (Facebook, Twitter, Instragram) (*skip question 21)*
- ☐ Zoom, Tumblr *(pass question 21)*
- ☐ Music festivals (e.g., technodance, electronic, rave) (*skip question 21)*
- ☐ Mass events (e.g., LGBT Pride march) *(skip question 21)*
- ☐ They were sexual partners I already knew (fuck buddies, friends with benefits). *(skip question 21)*
- ☐ Other(s):____*(skip question 21)*
- ☐ DK/NA *(skip question 21)*

21. Indicate how often you performed or attended the following activities in the last 6 months

(skip question 22)

	Never	1-2 times	3-4 times	5-10 times	More of 10 times	DK/NA
Going to a gay sauna	☐	☐	☐	☐	☐	☐
Entering a darkroom	☐	☐	☐	☐	☐	☐
Go to a Circuit festival party or similar	☐	☐	☐	☐	☐	☐
Attending a Pride event	☐	☐	☐	☐	☐	☐
Going to a gay discotheque	☐	☐	☐	☐	☐	☐
Attending private sex/chill out parties	☐	☐	☐	☐	☐	☐
Attending events or fetish clubs	☐	☐	☐	☐	☐	☐
Go cruising	☐	☐	☐	☐	☐	☐
Other events, specify: ________________	☐	☐	☐	☐	☐	☐

22. Have you ever had sex in exchange for money, gifts or favors?

a. Yes *(skip question 23)*
b. No *(skip question 24)*
c. DK/NA *(skip question 24)*

23. In the last 12 months, how many times have you charged for sex with a man?

By charging we mean that he gave you money, gifts or did favors in exchange for sex.

a. None *(skip question 24)*
b. 1 - 2 times *(pass question 24)*
c. 3 - 10 times *(pass question 24)*
d. 11 - 50 times *(pass question 24)*
e. More than 50 times *(pass question 24)*
f. DK/NA *(skip question 24)*

PART D. SUBSTANCE USE

24. **In the last 12 months, have you ever used any substance, including alcohol, Viagra, and other recreational drugs?**
    - *a.* Yes *(skip question 25)*
    - *b.* No *(skip question 28)*
    - *c.* DK/NA *(skip question 28)*

25. **When was the last time you used any of the following substances?**

    (skip question 26)

	Latest 30 days	Latest 6 Months	Latest 12 months	More than 12 months	Never	DK/NA
Alcohol	☐	☐	☐	☐	☐	☐
Sedatives or tranquilizers	☐	☐	☐	☐	☐	☐
Viagra® or similar	☐	☐	☐	☐	☐	☐
Popper	☐	☐	☐	☐	☐	☐
Ecstasy	☐	☐	☐	☐	☐	☐
MDMA	☐	☐	☐	☐	☐	☐
Methamphetamine	☐	☐	☐	☐	☐	☐
Mephedrone	☐	☐	☐	☐	☐	☐
GHB/L	☐	☐	☐	☐	☐	☐
Ketamine	☐	☐	☐	☐	☐	☐
Cocaine	☐	☐	☐	☐	☐	☐
Other recreational drugs	☐	☐	☐	☐	☐	☐

26. **Have you intentionally used drugs to have sex, which is known as being on a *Chemsex* session, or high, chillout, party?**
    - *a.* No, never *(skip question 28)*
    - *b.* Yes, in the last month *(skip question 27)*
    - *c.* Yes, in the last 6 months *(skip question 27)*
    - *d.* Yes, in the last 12 months *(skip question 27)*
    - *e.* Yes, more than 12 months ago *(skip question 27)*
    - *f.* DK/NA *(skip question 27)*

27. Have you ever slammed or slamming?

(slamming: the use of injectable drugs in sex sessions).

a. No, never *(skip question 28)*

b. Yes, in the last month *(skip question 28)*

c. Yes, in the last 6 months *(skip question 28)*

d. Yes, in the last 12 months *(skip question 28)*

e. Yes, more than 12 months ago *(skip question 28)*

f. DK/NA *(skip question 28)*

PART E. STIs AND TESTING

28. Why do you want to take the Mpox test (check ALL that apply)?

- ☐ Because I have had anal sex without a condom *(pass question 29).*
- ☐ Because I have had vaginal sex without a condom *(pass question 29).*
- ☐ Because I have had oral sex without a condom *(pass question 29).*
- ☐ Condom breakage or slippage *(skip question 29)*
- ☐ Regular control *(pass question 29)*
- ☐ Just to know my health status *(pass question 29)*
- ☐ I have had sexual intercourse with a person who has Mpox. *(skip question 29)*
- ☐ I have been in contact (non-sexual) with a person who has Mpox. *(skip question 29)*
- ☐ Because my partner asks me to *(pass question 29).*
- ☐ Before I stopped using condoms with my partner *(skip to question 29)*
- ☐ Other, specify:_________________________*(skip question 29)*

29. In the past 12 months, have you been diagnosed with any of these infections? *(check all that apply)*

- ☐ None *(skip question 30)*
- ☐ Syphilis *(skip question 30)*
- ☐ Chlamydia *(skip question 30)*
- ☐ Gonorrhea *(pass question 30)*
- ☐ Lymphogranuloma venereum (LGV) (*skip question 30)*
- ☐ Genital herpes *(skip question 30)*
- ☐ Human papillomavirus (HPV) (genital/anal warts or condylomas) *(skip question 30)*
- ☐ Mycoplasma genitalium *(pass question 30)*
- ☐ Hepatitis A *(skip question 30)*
- ☐ Hepatitis B *(skip question 30)*
- ☐ Hepatitis C *(skip question 30)*
- ☐ Other STI: specify___________*(skip question 30)*
- ☐ DK/NA*(skip question 30)*

30. **Have you been diagnosed with any enteric infections such as shigellosis, amebiasis and giardiasis, and Campylobacter coli bacterial gastroenteritis?**
    a. No, never *(skip question 31)*
    b. Yes, in the last month *(skip question 31)*
    c. Yes, in the last 6 months *(skip question 31)*
    d. Yes, in the last 12 months *(skip question 31)*
    e. Yes, more than 12 months ago *(skip question 31)*
    f. DK/NA *(skip question 31)*

31. **Have you ever been diagnosed with scabies?**
    a. No, never *(skip question 32)*
    b. Yes, in the last month *(skip question 32)*
    c. Yes, in the last 6 months *(skip question 32)*
    d. Yes, in the last 12 months *(skip question 32)*
    e. Yes, more than 12 months ago *(skip question 32)*
    f. DK/NA *(skip question 32)*
32. **The last time you were diagnosed with an STI, did you or your health care provider inform your recent sexual partners, last two months, that they also needed testing/treatment?**
    a. No, none of them *(skip question 33).*
    b. Yes, to some of them *(skip question 33).*
    c. Yes, to all of them *(skip question 33).*
    d. DK/NA *(skip question 33)*
33. **Are you HIV positive?**
    a. Yes *(skip question 36)*
    b. No *(skip question 34)*
    c. I do not know my HIV status *(pass question 34).*
    d. I don't want to answer *(skip question 34)*

PART F. PRE-EXPOSURE PROPHYLAXIS AND VACCINATION

34. **Do you take or have you taken PreP on a regular basis?**
    a. Yes *(skip question 35)*
    b. No *(skip question 36)*
    c. Ns/DK/NA *(skip question 36)*

35. **How long have you been taking PrEP?**
    a. Yes, in the last month *(skip question 36)*
    b. Yes, in the last 6 months *(skip question 36)*
    c. Yes, in the last 12 months *(skip question 36)*
    d. Yes, more than 12 months ago *(skip question 36)*
    e. DK/NA *(skip question 36)*

36. **Have you received the smallpox vaccine?**
    a. Yes, I was vaccinated MANY years ago (I Spain all those born before 1981 received the vaccines *(skip question 38).*
    b. Yes, I have received the vaccine in the last 12 months *(skip question 38).*
    c. No *(skip question 37)*
    d. DK/NA *(skip question 37)*
37. **Will you agree to be vaccinated for Mpox when the vaccine becomes available?**
    a. Very likely *(pass question 38)*
    b. Fairly likely *(pass question 38)*
    c. Neither very nor unlikely *(pass question 38).*
    d. Unlikely *(skip question 38)*
    e. Not likely *(pass question 38)*
    f. DK/NA *(skip question 38)*

PART G. RISK ASSESSMENT

38. **Imagine you were diagnosed with Mpox, would you isolate yourself for 21 days?**
    a. Very likely *(pass question 39)*
    b. Fairly likely *(pass question 39)*
    c. Neither very nor unlikely *(pass question 39).*
    d. Unlikely *(pass question 39)*
    e. Not likely *(pass question 39)*
    f. DK/NA *(skip question 39)*
39. **How concerned are you about Mpox?**
    a. A lot *(pass question 40)*
    b. Fairly *(pass question 40)*
    c. I do not care much or little *(pass question 40).*
    d. Little *(pass question 40)*
    e. Nothing *(pass question 40)*
    f. DK/NA *(skip question 40)*
40. **How likely do you think it is that you will get Mpox?**
    a. Very likely *(pass question 41)*
    b. Fairly likely *(pass question 41)*
    c. Neither very nor unlikely *(pass question 41).*
    d. Unlikely *(skip question 41)*
    e. Not likely *(pass question 41)*
    f. DK/NA *(skip question 41)*
41. **Do you take off your shirt or T-shirt when you are partying in a club or disco?**
    a. Very likely *(pass question 42)*
    b. Fairly likely *(pass question 42)*
    c. Neither very nor unlikely *(pass question 42).*
    d. Unlikely *(pass question 42)*
    e. Not likely *(pass question 42)*
    f. DK/NA *(skip question 42)*

42. Do you plan to change your behavior in the next 3 months? Please rate the following situations: *(skip question 43)*

	YES	Probably YES	It could be YES or NO	Probably NO	NO	NS/NC
Have less sexual partners	▯	▯	▯	▯	▯	▯
Have less sex	▯	▯	▯	▯	▯	▯
Avoid crowds	▯	▯	▯	▯	▯	▯
Avoid dense locations	▯	▯	▯	▯	▯	▯
Avoiding pool parties	▯	▯	▯	▯	▯	▯
Check your sexual partner's body for signs of Mpox.	▯	▯	▯	▯	▯	▯
Talking about the risk of Mpox with your sexual partner	▯	▯	▯	▯	▯	▯

PART H. EVALUATION OF EXPERIENCE

43. How much do you agree or disagree with the following statement: "I am confident that I have collected the pharyngeal swab well"?

a. Strongly agree *(pass question 44)*
b. Strongly Agree *(pass question 44)*
c. Neither agree nor disagree *(skip to question 44).*
d. Strongly Disagree *(skip question 44)*
e. Strongly Disagree *(skip question 44)*
f. DK/NA *(skip question 44)*

44. How much do you agree or disagree with the following statement: "I am confident that I have collected the <u>anal</u> specimen well"?

a. Strongly agree *(pass question 45)*
b. Strongly Agree *(pass question 45)*
c. Neither agree nor disagree *(pass question 45)*
d. Strongly Disagree *(skip question 45)*
e. Strongly Disagree *(skip question 45)*
f. DK/NA *(skip question 45)*

45. How easy or difficult did you find it to perform the <u>pharyngeal</u> self-sampling for Mpox?

a. Very easy *(pass question 46)*
b. Fairly easy *(pass question 46)*
c. Neither easy nor difficult *(pass question 46).*
d. Quite difficult *(pass question 46)*
e. Very difficult *(pass question 46)*
f. DK/NA *(skip question 46)*

46. How easy or difficult did you find it to perform the <u>anal</u> self-sampling for Mpox?

a. Very easy *(pass question 47)*
b. Fairly easy *(pass question 47)*
c. Neither easy nor difficult *(pass question 47).*
d. Quite difficult *(pass question 47)*
e. Very difficult *(pass question 47)*
f. DK/NA *(skip question 47)*

47. What is your assessment of the experience of having the Mpox self-sampling?

a. Very satisfied *(skip question 48)*
b. Fairly satisfied *(skip question 48)*
c. Neither satisfied nor dissatisfied *(skip question 48)*
d. Fairly dissatisfied *(skip question 48)*
e. Very dissatisfied *(skip question 48)*
f. DK/NA *(skip question 48)*

48. Do you agree or disagree with this statement: "I would repeat the Mpox self-sampling in the future"?

a. Strongly agree *(pass question 49)*
b. Strongly Agree *(pass question 49)*
c. Neither agree nor disagree *(pass question 49)*
d. Strongly Disagree *(skip question 49)*
e. Strongly Disagree *(skip question 49)*
f. DK/NA *(skip question 40)*

49. Do you agree or disagree with this statement: "I would recommend Mpox self-sampling to a friend"?

a. Strongly agree *(pass question 50)*
b. Strongly Agree *(pass question 50)*
c. Neither agree nor disagree *(pass question 50)*
d. Strongly Disagree *(pass question 50)*
e. Strongly Disagree *(skip question 50)*
f. DK/NA *(skip question 50)*

50. How much do you agree or disagree with the following statement: "I consider self-sampling for Mpox detection to be a good strategy to screen for Mpox "?

a. Strongly agree *(pass question 51)*
b. Fairly Agree *(pass question 51))*
c. Neither agree nor disagree *(pass question 51)*
d. Strongly Disagree *(skip question 51)*
e. Strongly Disagree *(skip question 51)*
f. DK/NA *(skip question 51)*

51. What advantages do you think this type of test has? (Please indicate as many as you consider)

- ☐ Privacy and confidentiality *(skip question 52).*
- ☐ More convenience as you don't have to go to the medical center *((pass question 52).*
- ☐ That the test is free of charge. *(pass question 52)*
- ☐ You do not need a prescription. *(skip question 52)*
- ☐ You don't have to explain yourself to anyone *(pass question 52).*
- ☐ Contribute to standardizing the Mpox test *(pass question 52).*
- ☐ Allows me to take control of my health with respect to Mpox *(skip question 52).*
- ☐ Others:_____________________________________*(skip question 52)*
- ☐ DK/NA *(skip question 51)*

52. What disadvantages do you consider this type of test to have? (Point out as many as you consider)

- ☐ That the test requires the introduction of a swab orally *(skip question 53).*
- ☐ That the test requires the introduction of a swab via the rectum *(skip question 53).*
- ☐ That you don't have the result at the moment *(pass question 53).*
- ☐ That you have no emotional support when receiving the result *(skip question 53).*
- ☐ The time to receive the result is too long *(skip question 53).*
- ☐ There is a risk that the sample may be lost during shipment *(skip question 53).*
- ☐ Others:____*(skip question 53)*
- ☐ DK/NA *(skip question 53)*

53. **If you had to repeat the Mpox test, would you prefer: (Check all that you consider)**

- ☐ Go to a health center (Clinic, Hospital, CAP, etc) (*skip question 54).*
- ☐ Go to a community center/NGO *(skip question 54)*
- ☐ Self-sampling at home *(pass question 54)*
- ☐ DK/NA *(skip question 54)*

54. **Comments:**

FIN ☺

THANK YOU VERY
MUCH!!!
