# Supplementary material for: Self-sampling monkeypox virus testing in high-risk populations, asymptomatic or with unrecognized Mpox, in Spain
Source: Nat Commun. 2023 Oct 2;14:5998. doi: 10.1038/s41467-023-40490-9 (PMC10545734; doi:10.1038/s41467-023-40490-9)
Supplement: Supplementary file 3 — Description of Additional Supplementary Files [file 41467_2023_40490_MOESM3_ESM.pdf]

Description of Additional Supplementary Files

File Name: Supplementary Data 1
Description: Sequences of primers used and information of utilized reagents
